# Comparison of Various GAN‐Based Bone Suppression Imaging for High‐Accurate Markerless Motion Tracking of Lung Tumors in CyberKnife Treatment

**DOI:** 10.1111/1759-7714.70014

**Published:** 2025-02-24

**Authors:** Zennosuke Mochizuki, Masahide Saito, Toshihiro Suzuki, Koji Mochizuki, Hikaru Nemoto, Hiroshi Onishi, Hiroshi Takahashi

**Affiliations:** ^1^ Department of Radiology Kasugai General Rehabilitation Hospital Yamanashi Japan; ^2^ Department of Radiology University of Yamanashi Chuo Japan

**Keywords:** deep learning, generative adversarial networks (GANs), lung cancer, radiotherapy, stereotactic radiotherapy

## Abstract

Stereotactic body radiation therapy (SBRT) is a highly effective treatment for lung cancer; however, challenges arise from tumor motion induced by respiration. The CyberKnife system, incorporating both fiducial‐based and fiducial‐free tracking modalities, aims to mitigate these challenges, yet tumor recognition can be compromised by overlapping bone structures. This study introduces a novel bone suppression imaging technique for kilovolt X‐ray imaging using generative adversarial networks (GANs) to enhance tumor tracking in SBRT by reducing interference from bony structures. Computed tomography (CT) images, both with and without bone structures, were generated using a four‐dimensional extended cardiac‐torso phantom (XCAT phantom) across 56 cases. X‐ray projections were captured from left and right oblique 45° angles and divided into nine segments, producing 1120 images. These images were processed through six pre‐trained GAN models—CycleGAN, DualGAN, CUT, FastCUT, DCLGAN, and SimDCL—yielding bone‐suppressed images on the XCAT phantom (BSI_phantom_). The resulting images were evaluated against bone‐shadow‐free images using structural similarity index measure (SSIM), peak signal‐to‐noise ratio (PSNR), and Frechet inception distance (FID). Additionally, bone‐suppressed images (BSI_patient_) were derived from 1000 non‐simulated patient images. BSI_phantom_ images achieved SSIM and PSNR values of 0.96 ± 0.02 and 36.93 ± 3.93, respectively. SimDCL exhibited optimal performance with an FID score of 68.93, indicative of superior image generation quality. This GAN‐based bone suppression imaging technique markedly improved image recognition and refined dynamic tumor tracking, enhancing the accuracy and efficacy of SBRT.

## Graphical abstract

A novel bone suppression imaging technique employing generative adversarial networks enhances tumor tracking in stereotactic body radiation therapy (SBRT) for lung cancer. By mitigating interference from osseous structures in X‐ray images, this technique improves tumor recognition and dynamic tracking capabilities. The SimDCL demonstrated optimal performance, yielding substantial improvements in image quality and tracking precision, thereby significantly augmenting the efficacy of SBRT.

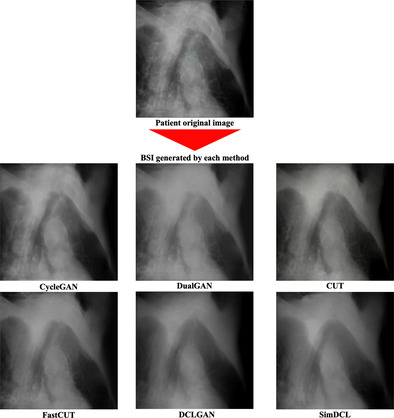

## 1 Introduction

Stereotactic body radiation therapy (SBRT) is widely used for lung cancer treatment, showing good outcomes [1]. SBRT combines multidirectional, precise irradiation to improve local tumor control and reduce adverse events in nearby normal tissue. One challenge is tumor movement due to respiration. Various countermeasures have been developed to administer precise doses and minimize impact on normal tissue [2, 3].

The CyberKnife (CK) system (G4, version 10.5, Accuray Inc.) integrates a robotic‐positioned linear accelerator, an image‐guided system, and respiratory tracking systems. CK has two respiratory tracking systems: the fiducial‐based target tracking system (FTTS) and the Xsight lung tracking system (XLTS) [4]. XLTS, a fiducial‐free real‐time tracking system, is used to irradiate lung tumors that move with respiration [5]. Retrospective studies show good 5‐year local control and overall survival, 91% and 75%, respectively [6, 7]. The target locating system (TLS) uses orthogonal X‐ray imagers to track moving targets by modeling the correlation between targets and external LED markers on the patient's chest [8]. This system tracks the treatment area in real‐time with irradiation error under 1 mm [9, 10]. While systems like Varian TrueBeam and Elekta Agility offer advanced image‐guided and motion management technologies, CK excels with its fiducial‐free real‐time tracking [11, 12].

Generally, XLTS aligns live X‐rays with their corresponding digitally reconstructed radiography (DRR) by utilizing pixel intensities derived exclusively from the tumor and its surrounding soft tissues. Tumors that are smaller or obscured by dense anatomical structures (such as the mediastinum, heart, rib cage, or spine) cannot be consistently detected or tracked with current XLTS algorithms, highlighting the need for enhanced detection techniques [13, 14]. To resolve this, six generative adversarial network (GAN) approaches for bone suppression imaging were implemented and compared. Unsupervised training often proves unstable due to multiple valid domain mappings, while cycle‐consistent models fail to handle significant geometric alterations. To overcome these limitations, GANs integrating contrast and dual learning were employed, enabling high‐quality transformations across distinct domains. This strategy enhances tumor visibility and tracking accuracy in complex cases, potentially exceeding the performance of existing systems.

In recent years, GANs have shown state‐of‐the‐art performance in many image‐processing tasks [15]. Within the domain of image‐to‐image conversion, there are two fundamental categories: supervised [16, 17, 18] and unsupervised learning [19, 20, 21]. Conditional GAN [22] and pix2pix [16] rely on paired data, while alternatives like Cycle‐consistency GAN (CycleGAN) [23], DualGAN [19, 24], Contrastive Learning for Unpaired Image‐to‐Image Translation (CUT) [25], FastCUT [25], Dual Contrastive Learning GAN (DCLGAN) [26], and Simultaneous Dual Contrastive Learning (SimDCL) [26] do not. CycleGAN eliminates the need for paired training data, using a ResNet‐based generator and a PatchGAN classifier. DualGAN, for asymmetric transformations, incorporates dual loss functions. CUT leverages partial contrastive learning for efficient image transformation, while FastCUT is a quicker iteration. DCLGAN, designed for unpaired data, addresses adversarial challenges with dual contrast learning, while SimDCL improves inversion performance. GANs also aid in computer‐aided diagnosis by removing obstructive bone structures in lung imaging [27], as well as reducing artifacts in both computed tomography (CT) and cone beam CT scans [28, 29, 30].

We previously reported on image recognition through bone suppression imaging technology based on CycleGAN, enabling high‐precision motion tracking irradiation [31]. While systems like CycleGAN demonstrate significant potential in image transformation, their inability to preserve geometric fidelity can undermine tumor tracking reliability, especially in complex cases with overlapping structures like bones. Models such as SimDCL, which prioritize both image quality and computational efficiency, offer a promising solution [32]. This study evaluates various GAN models with distinct algorithms to analyze differences in tumor detection performance based on image quality and generation time. By comparing these advanced models with traditional methods, it seeks to enhance tumor visibility during radiation therapy and improve the precision of real‐time tracking.

## 2 Methods

**FIGURE 1:**
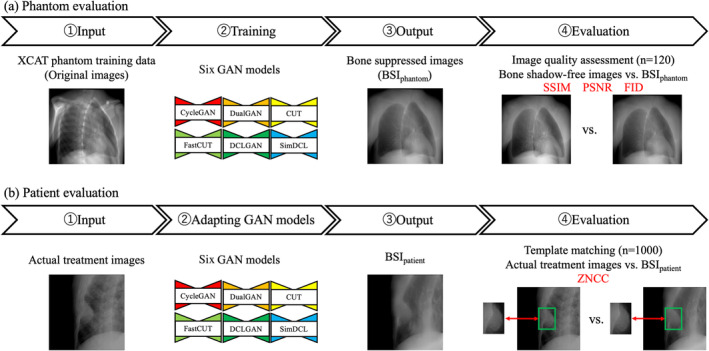
Work‐flow of the bone suppression and evaluation framework. (a) Six pre‐trained GAN models generated bone‐suppressed images (BSI_phantom_) from original images, which were validated against test data. (b) BSI_patient_ images were then generated from actual CK treatment images, and detection effectiveness was evaluated using template matching.

Six pre‐trained GAN models were employed to input original images, generating bone suppressed images (BSI_phantom_). These BSI_phantom_ images were subsequently compared with test data for validation. Next, BSI_patient_ were generated from the images used for the actual CK treatment, and the effectiveness of deep learning was evaluated by template matching for each of the actual treatment images and BSI_patient_. The workflow is illustrated in Figure 1. During training, key hyperparameters were set to optimize model performance. Table 1 provides an overview of the selection criteria and features for each GAN model. Lr represents the learning rate, while epochs represent the number of epochs. The selection criteria highlight specific attributes considered when choosing each model. In conventional deep learning, a smaller loss generally indicates that the model is learning effectively. Typically, once the loss decreases to a certain threshold and plateaus, it is considered an appropriate point to terminate training. However, in GAN training, if one side's loss drops too quickly, it signifies a failure in learning. In other words, the balance between the Generator and Discriminator losses is critical in GANs. In this study, we successfully adjusted this balance by modifying the Discriminator network, such as reducing its size and increasing the use of dropout. The appropriate setting of these hyperparameters significantly influenced the quality of bone‐suppressed images generated by the GAN models, ensuring that outputs were accurate and clinically useful. This careful consideration ensures that the chosen GAN models meet technical requirements and provide a reliable solution for enhancing medical imaging applications.

**TABLE 1:** Selection process and hyperparameters of GAN models.

Models	Selection criteria	Lr	Batch size	Epochs
CycleGAN	Bidirectional image translation for unpaired images using cycle consistency.	0.0005	2	400
DualGAN	Bidirectional translation with two adversarial networks for unpaired data.	0.0001	4	400
CUT	Efficient image translation with contrastive learning from unpaired data.	0.0002	3	400
Fastcut	Faster image translation with contrastive learning at the patch level.	0.0002	5	400
DCLGAN	Improved translation accuracy by applying contrastive learning bidirectionally.	0.0005	1	400
SimDCL	Simple and efficient image translation with basic contrastive learning.	0.0006	1	400

### 2.1 Data

#### 2.1.1 4D Extended Cardiac‐Torso Phantom (XCAT Phantom) Images

**FIGURE 2:**
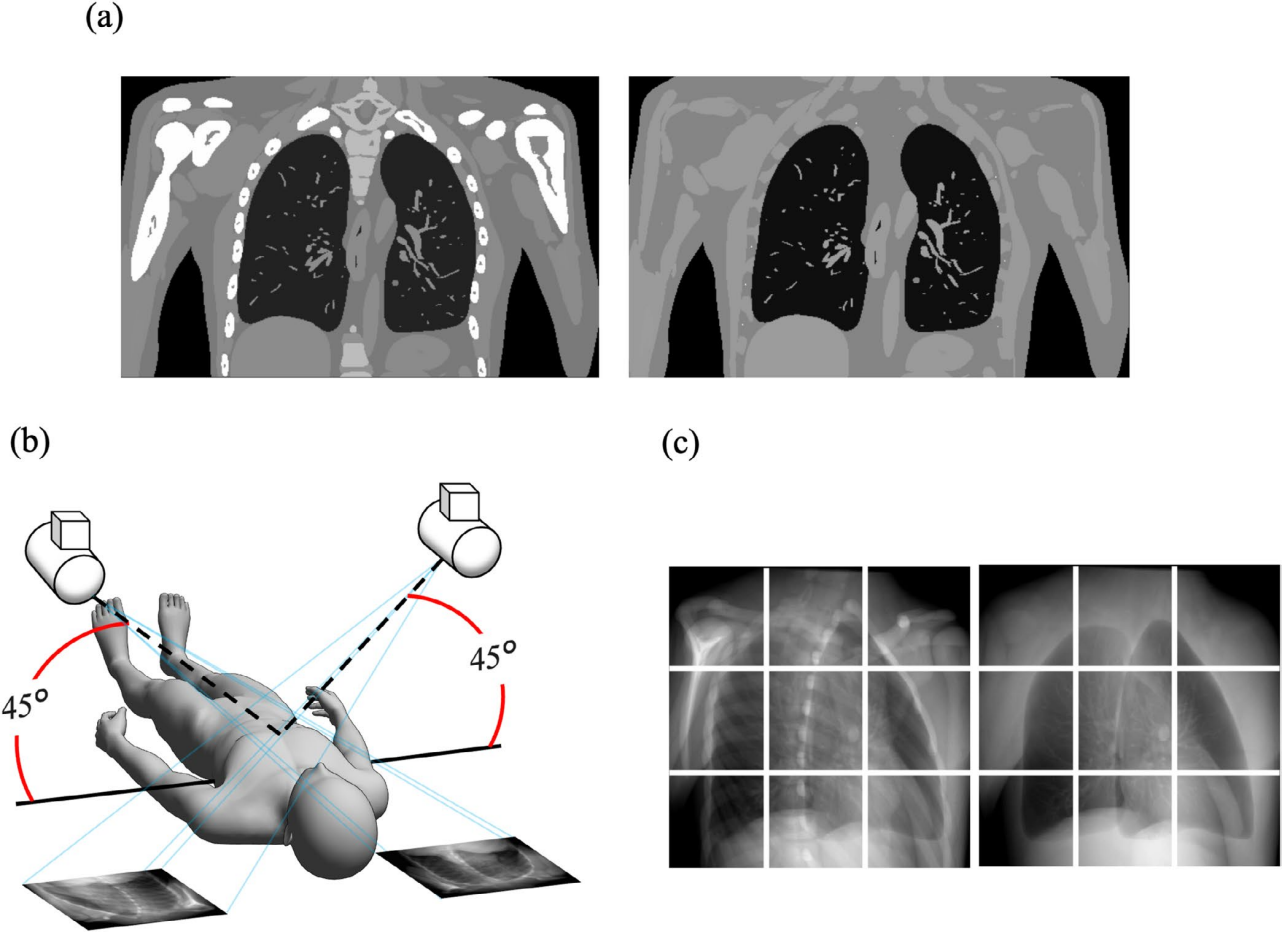
Creation of original images and bone shadow‐free images generated from the XCAT phantom. (a) Original images (L)/bone shadow‐free images (R). (b) Pseudo‐projecting the XCAT phantom. (c) Projected original images (L)/bone shadow‐free images (R) as in (b) were divided into nine segments.

We first created images from XCAT phantom (Duke University, Durham, North Carolina) [33, 34] for deep‐learning datasets. The XCAT phantom, a digital anthropomorphic phantom image database, is based on the National Library of Medicine's human anatomy database. It uses non‐uniform rational b‐spline surfaces and combines voxelized and mathematical approaches for realistic simulated imaging with detailed, flexible organs, allowing for anatomical variation and deformation [34, 35, 36]. Though positioned off‐center, it mimics the rotation and shifts seen in patient CT images. We used 56 samples from this phantom, differing in age, sex, ethnicity, height, and weight. The dataset included 56 individuals aged 18–78 years, weight 52–120 kg, height 153–190 cm, and BMI 18–39 kg/m^2^. The dxcat2 code of XCATv2 software obtained images with and without bony structures, as shown in Figure 2a. Settings included a bone size parameter of one, pixel and slice width of 0.03 cm, and an array size of 512 × 512.

Next, to create 2D CK images, we used 3D Slicer software (version 4.11, MIT, Massachusetts, USA) to create 45° images, as shown in Figure 2b. Parameters included view‐up vector (x,y,z) = (−1,0,45), normal vector (x,y,z) = (45,−45,0), and isocenter position (x,y,z) = (0,0,0). These were divided into nine segments, as shown in Figure 2c. This process created 1120 images, of which 120 were used for testing. Optimal model performance, with reduced training loss, was observed when splitting training and testing sets on a per‐patient basis, minimizing data leakage and allowing better generalization across patients.

#### 2.1.2 Treatment Images

To evaluate the model, treatment images were obtained from 50 patients with metastatic (tumor size 8–64 mm) and primary lung cancer (tumor size 10–62 mm) undergoing stereotactic radiotherapy at our center between February 2020 and January 2022. Forty patients were male and 10 were female, aged 46–93 years (average age, 74.14). No specific selection criteria were applied to tumor types, resulting in a diverse mix of solid, part‐solid, and ground‐glass nodules (GGNs). Patients were consecutively selected based on their treatment schedules to mitigate selection bias and ensure a representative sample. Stereotactic radiotherapy using CK synchronous respiratory tracking technology was performed. All patients were treated with the CK system using 2‐view XLTS. CK DRR images have 512 × 512 pixels with a pixel size of 0.4 mm and 12‐bit intensity values (stored as 16‐bit integers, but the dynamic range is 12 bits).

### 2.2 Evaluation Methods

BSI_phantom_ were compared to bone shadow‐free images from the test data, and the similarity was calculated. To accurately assess the quality of images generated by models, quantitative evaluation metrics are indispensable. Beyond subjective evaluation by human observers, it is crucial to implement objective methods for measuring image quality. Accurately gauging the quality of generated images remains a persistent challenge [37]. For instance, quantitative metrics such as Frechet inception distance (FID) may not fully capture the realism or naturalness of the images [38, 39]. Therefore, it is recommended to combine these metrics with visual inspections and subjective evaluations for a more comprehensive assessment. We evaluated the models with three different objective image quality metrics, the structural similarity index measure (SSIM) [40], peak signal‐to‐noise ratio (PSNR) [41], and FID [42]. These evaluations were conducted individually for each image, as illustrated in Figure 1.

SSIM evaluates image quality based on structural information, as described by Equations (1) and (2). Equation (1) calculates the SSIM for each block, while Equation (2) computes the average SSIM across all blocks.

(1) $$\text{SSIM}\left(x,y\right)=\frac{\left(2\mu_{x}\mu_{y}+C_{1}\right)\left(2\sigma_{\mathrm{xy}}+C_{2}\right)}{\left(\mu_{x}^{2}+\mu_{y}^{2}+C_{1}\right)\left(\sigma_{x}^{2}+\sigma_{y}^{2}+C_{2}\right)}$$

(2) $$\text{MSSIM}=\frac{1}{M}\sum_{j=1}^{M}\text{SSIM}\left(x_{j},y_{j}\right)$$

Here, x and y are the individual blocks of the reference and test images, respectively, and μ is the average per block of the Gaussian filtered image. Similarly, $\sigma^{2}$ refers to the variance per block of the Gaussian filtered image: $C_{1}$ and $C_{2}$ are constants, ${\left(0.01\times 255\right)}^{2}$ and ${\left(0.03\times 255\right)}^{2}$, respectively. M is the number of blocks.

PSNR is an index obtained by the ratio of the mean squared errors (MSEs) of the reference image and the test image to the maximum grayscale value (PS = 255). The PSNR and MSE are shown in Equations (3) and (4), respectively.

(3) $$\text{PSNR}=10\;\log_{10}\left(\frac{{\mathrm{PS}}^{2}}{\mathrm{MSE}}\right)$$

(4) $$\mathrm{MSE}=\frac{1}{N}\sum_{1}^{N}{\left(x_{i}-y_{i}\right)}^{2}$$

Here, $x_{i}$ represents the reference image, $y_{i}$ represents the grayscale value of the test image, and N represents the total number of images.

FID is a quantitative metric that employs an inception network to convert reference and test images into feature vectors, retaining their higher‐dimensional information. By assuming a Gaussian distribution for these feature vectors, the mean and covariance matrices are computed, as presented in Equation (5).

(5) $$\mathrm{FID}={\left|\left|\mu_{X}-\mu_{Y}\right|\right|}^{2}+\mathrm{Tr}\left(\Sigma_{X}+\Sigma_{Y}-2\sqrt{\Sigma_{X}\Sigma_{Y}}\right)$$

Here, μ is the mean, Σ is the covariance, and Tr is the sum of the diagonal elements.

Next template matching was performed on each treatment image and BSI_patient_ to calculate the zero‐mean normalized cross‐correlation (ZNCC). In this study, the entire image served as the search area to capture the tumor contour. Template matching, a fundamental detection technique, identifies the target location by comparing the input image with a pre‐defined template. ZNCC quantifies similarity by adjusting for lighting variations, with values ranging from −1 to 1, where 1 denotes a perfect match. After processing 1000 treatment images through the GAN to BSI_patient_, template matching was applied to calculate ZNCC.

As a preprocessing step, no alterations were made to the training, test, or treatment images; however, refinements, including noise reduction and adjustments for size and angle, were applied to enhance accuracy.

### 2.3 Statistical Analysis

All statistical analyses were performed using SPSS Statistics for Windows (version 27.0, IBM Corp., Armonk, NY). The results are reported as the mean ± SEM. Initially, the Shapiro–Wilk test was employed to evaluate the normality of the quantitative data. Given the non‐normal distribution of the data, the Friedman test with the Bonferroni correction was utilized to analyze the dependent data across six groups. Subsequently, the ZNCC between the BSI_patient_ and the actual treatment images (reference) was compared, employing the Wilcoxon rank‐sum test for significance testing. Differences were considered statistically significant at *p* < 0.05.

### 2.4 Computational Environment and Programming Language

The computational setup employed for this study comprised a CPU: Intel Core i7‐10 700 clocked at 3.8GHz, GPU: NVIDIA GeForce RTX 2080 SUPER with 8GB of memory, and the programming language Python version 3.9.18.

## 3 Results

**FIGURE 3:**
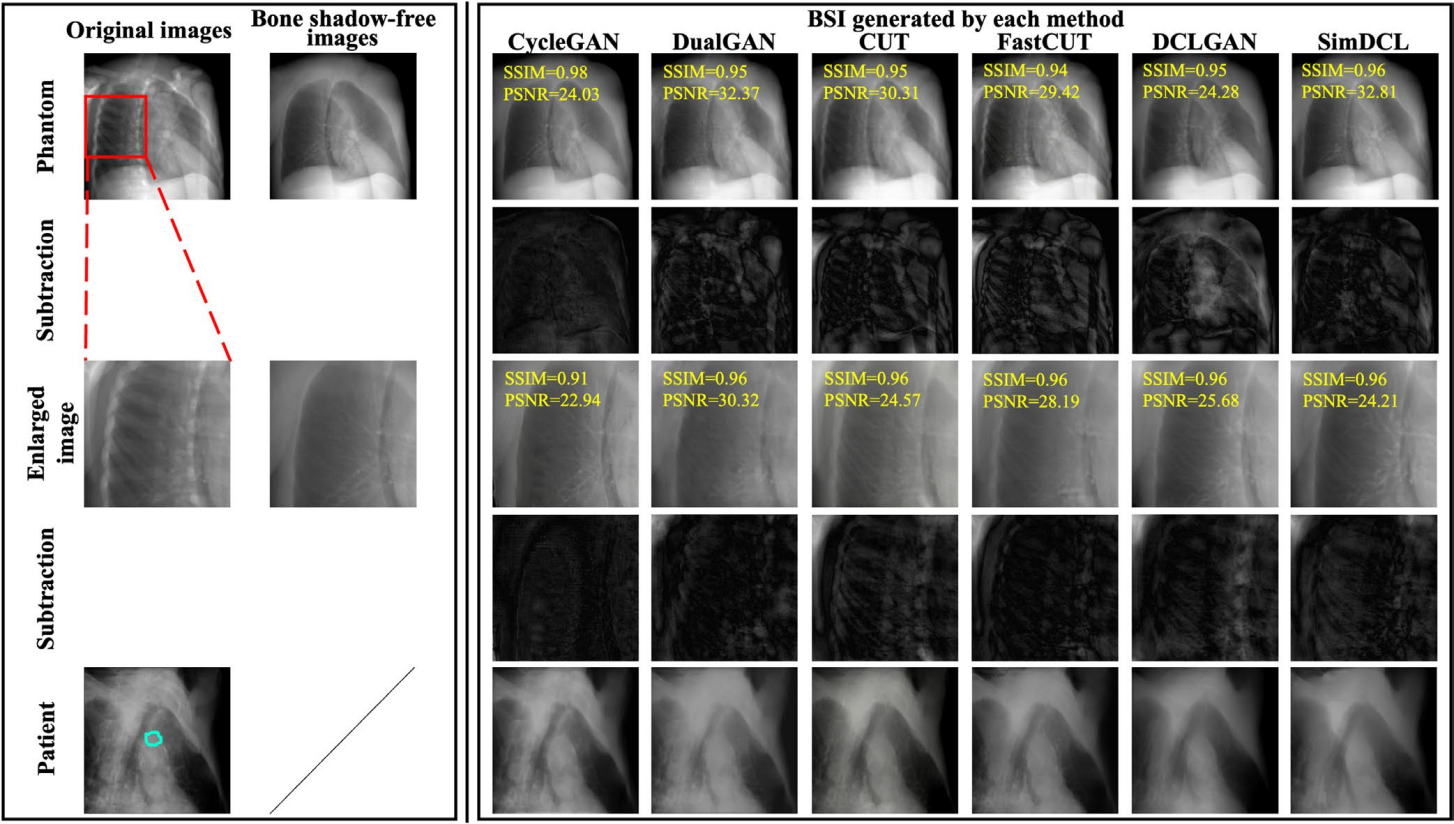
Examples between the test data and BSI. In the first column from the left, the original XCAT phantom images are shown at the top, with the bone shadow‐free images directly adjacent. The second row consists of subtraction images between the bone shadow‐free images and BSI_phantom_. While these subtraction images are essential for visualizing the differences, it can be difficult to clearly interpret them due to visibility issues. Therefore, contrast adjustments were applied to improve the visual representation of differences. The middle row contains magnified sections of the original images, while the fourth row illustrates the subtraction images corresponding to these magnified sections, again comparing the bone shadow‐free images with BSI_phantom_. The bottom row showcases BSI_patient_, highlighting the results generated through various methodologies. Additionally, the blue line in the treatment images denotes the tumor tracking volume (TTV). All images were adjusted to the window level 30/window width 330. All images were adjusted to the window level 30/window width 330.

Figure 3 highlights examples from the BSI_phantom_ and BSI_patient_, illustrating the comparison between bone shadow‐free images and those generated by the models. Figure 3 includes difference images, showing the subtraction of bone shadow‐free images from BSI_phantom_ images. The removal of bone shadows was essential for enhancing the clarity of tumor contours, which is particularly important for accurate stereotactic radiotherapy planning. After applying the GAN models, the bone intensities were significantly suppressed, while the tumor structures were preserved—an essential aspect for achieving successful radiotherapy outcomes. The ability to maintain this balance between bone removal and tumor preservation is crucial for the clinical application of these models.

**FIGURE 4:**
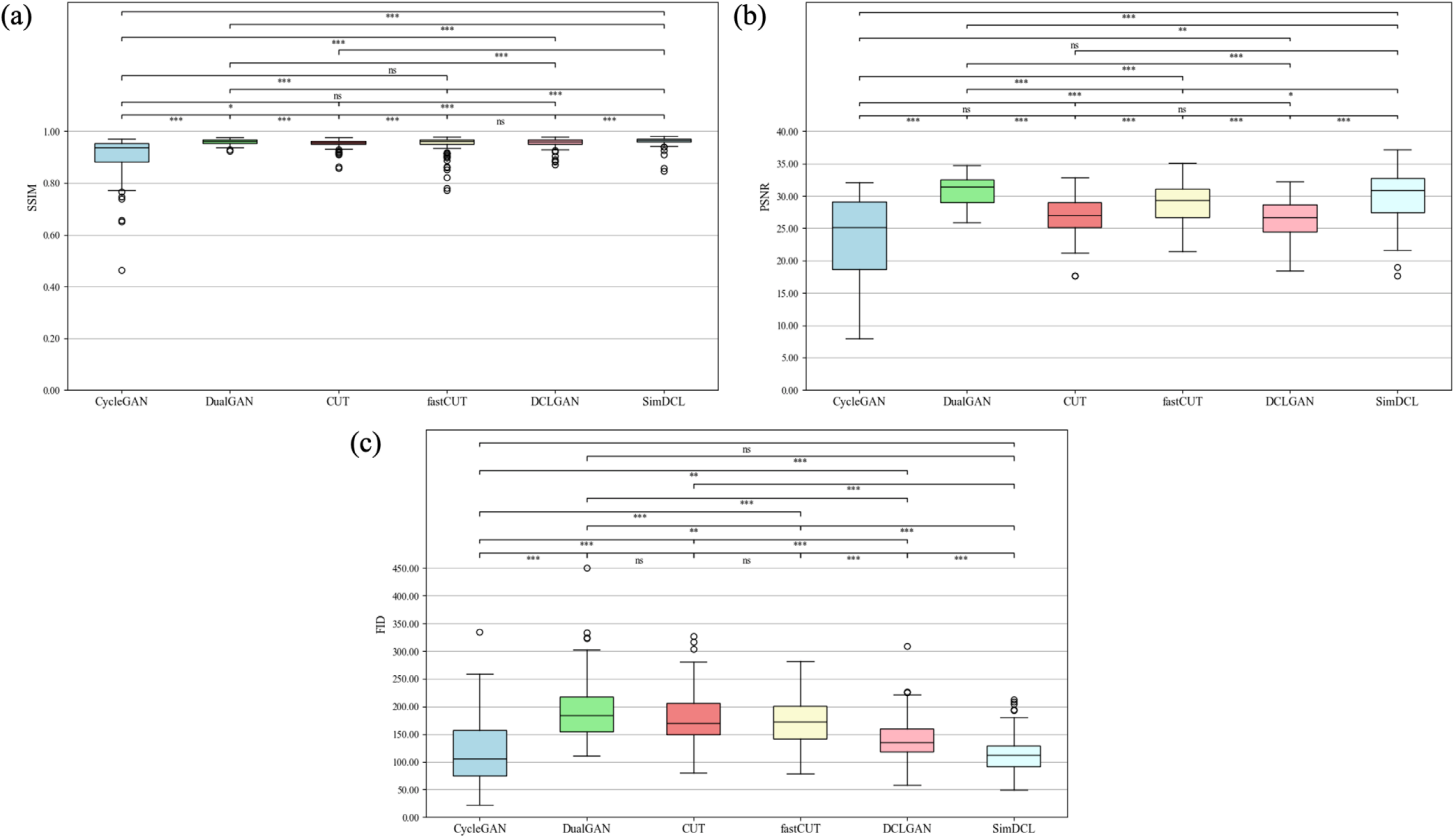
SSIM, PSNR, and FID metrics of the generated images from various GAN models. These metrics are illustrated through box plots for each model. In these plots, the central line denotes the median, while the upper and lower edges of the box signify the third (Q3) and first (Q1) quartiles, respectively. The whiskers extend to the minimum and maximum data points within 1.5 times the interquartile range (IQR) from Q1 and Q3, with any data points outside this range identified as outliers and marked individually. At the top of each plot, bars illustrate multiple model comparisons, with statistical significance indicated by *** for *p* < 0.001, ** for *p* < 0.01, * for *p* < 0.05, and “ns” for non‐significant results. This visual representation facilitates a clear comparison of the performance variations among the GAN models.

Table 2 and Figure 4 show the SSIM and PSNR values for the six GAN models. SSIM assesses the visual similarity between the generated and reference images, focusing on structural elements like luminance and contrast. A higher SSIM value indicates greater resemblance to the reference image, which is essential for preserving clinically relevant details after bone suppression. PSNR measures the ratio of signal to noise, reflecting image fidelity, with higher values signifying reduced noise and superior quality. The BSI_phantom_ model exhibited the highest SSIM (0.96 ± 0.02) and PSNR (36.93 ± 3.93), indicating optimal image integrity preservation during bone shadow removal. These elevated values suggest that BSI_phantom_ generated images closely match the reference, retaining critical anatomical details. The significant differences observed among the six models (SSIM: H(5) = 448.10, PSNR: H(5) = 311.17, *p* < 0.05) further underscore the superior image quality of BSI_phantom_.

**TABLE 2:** Objective image quality index between bone shadow‐free images and BSI_phantom_.

Metrics	CycleGAN	DualGAN	CUT	FastCUT	DCLGAN	SimDCL
SSIM	0.90 ± 0.06	0.90 ± 0.01	0.95 ± 0.02	0.95 ± 0.03	0.96 ± 0.02	0.96 ± 0.02
PSNR	31.54 ± 4.48	30.94 ± 2.22	33.94 ± 3.08	35.83 ± 3.00	33.43 ± 3.12	36.93 ± 3.93
FID	119.45 ± 59.28	193.45 ± 55.10	179.92 ± 49.45	173.23 ± 43.14	141.26 ± 38.09	114.52 ± 32.60

*Note:* The Friedman test was employed to evaluate the differences in metrics among the six sets of images; Figure 4 presents the detailed comparisons between each model.

The evaluation of FID, a metric that assesses the quality of generated images by comparing their statistical similarity to reference images, provided valuable insights. FID considers the mean and covariance of feature vectors extracted from the images, offering a robust assessment of image quality within a GAN framework. Lower FID scores indicate better alignment between the generated and reference distributions, signifying more realistic images. Among the models, SimDCL achieved the lowest FID score (114.52 ± 32.60), demonstrating its ability to generate the most realistic bone‐suppressed images. This result is crucial, as a low FID score enhances clinical applicability, with realistic imaging being vital for accurate tumor assessment and treatment planning. SimDCL demonstrated superior performance in this regard, highlighting its potential to improve accuracy in clinical workflows.

Template matching between the generated BSI_patient_ images and actual treatment images was performed using ZNCC to quantify the similarity. ZNCC is a powerful metric for assessing how well two images align, particularly when comparing templates to regions of interest, such as the tumor areas. A higher ZNCC value indicates better alignment, which is essential to ensure that the generated images can be reliably used in clinical settings. Table 3 shows that the CUT model achieves the highest ZNCC value (0.789 ± 0.159), demonstrating superior performance in aligning the generated images with real patient data. Significant differences (*p* < 0.05) were noted between the real patient images and those generated by several models (CycleGAN, CUT, FastCUT, DCLGAN, and SimDCL), indicating that these models effectively replicated the tumor's location and shape after bone suppression. This high ZNCC value is vital for clinical applications, where precise tumor localization is critical for treatment targeting.

**TABLE 3:** ZNCC value using template matching with actual treatment images and BSI_patient_.

	Actual treatment images (reference)	CycleGAN	DualGAN	CUT	FastCUT	DCLGAN	SimDCL
Number of images identified correctly	767	800	704	804	824	713	774
(%)	(76.7)	(80.0)	(70.4)	(80.4)	(82.4)	(71.3)	(77.4)
ZNCC	0.763 ± 0.136	0.773 ± 0.143*	0.775 ± 0.150	0.789 ± 0.159*	0.785 ± 0.160*	0.769 ± 0.158*	0.777 ± 0.161*
Speed (ms/image)	94.7 ± 0.0	66.0 ± 0.0	67.8 ± 0.0	46.0 ± 0.0	52.0 ± 0.0	18.0 ± 0.0	16.7 ± 0.0

* *p* < 0.05 significantly different compared with the reference.

In addition to image quality, the speed of image generation is a critical factor, particularly in real‐time clinical applications. Speed, measured in milliseconds per image (ms/image), indicates the efficiency with which a model can produce bone shadow‐free images. Rapid image generation is essential for real‐time use, such as during treatment planning or intraoperative procedures. The average generation speed across models was 94.7 ± 0.013 ms/image, demonstrating their ability to rapidly produce high‐quality images. The ability of the models to generate images swiftly without compromising quality underscores their potential for clinical integration.

The clinical evaluation, encompassing 50 patients with metastatic and primary lung cancer, emphasized the practical relevance of the findings. The variability in tumor size (8–64 mm) and location (central and peripheral) poses challenges for image generation models. Additionally, respiratory motion (2–20 mm) further complicates the process, as tumors shift during imaging, hindering accurate bone shadow suppression. The models' capacity to adapt to these dynamic conditions is crucial for their clinical applicability. Precise tumor alignment and visualization are vital for effective radiotherapy, especially for tumors in regions affected by significant respiratory motion. The strong performance of models such as SimDCL under these conditions demonstrates their potential to improve treatment accuracy and patient outcomes.

## 4 Discussion

The precision in identifying tumors and the robustness of the system are influenced by the confluence of tumors and bone shadows in TLS. We advocated the utilization of GANs to acquire the proficiency to attenuate the intensity of bone shadows on TLS. Experimental evaluations conducted on the XCAT phantom datasets substantiated the efficacy of the framework developed for this purpose. Furthermore, our research evolved from the antecedent CycleGAN methodology, proffering solutions to dilemmas arising from the intersection of bone shadows and tumors, as well as challenges associated with image generation speed, through a comparative exploration of various GANs.

Unlike previous studies, Bae et al. explored the comparative efficacy of deep learning‐based bone suppression and dual‐energy bone subtraction for pulmonary nodule detection, highlighting the critical role of bone suppression techniques [43]. Their findings demonstrated significant improvements in nodule detection, particularly when nodules overlapped with osseous structures. This aligns with our findings, where models like SimDCL effectively suppressed bone shadows while preserving tumor architecture, despite our primary focus on radiotherapy rather than pulmonary imaging. Similarly, the SFRM‐GAN developed by Rani et al. for bone suppression in chest radiographs shares parallels with our work [44]. By incorporating advanced loss functions, including Sobel and Perceptual loss, their model outperformed conventional methods in PSNR and SSIM, demonstrating the strengths of GANs in image denoising and quality preservation. Our study corroborates this, with SimDCL achieving superior SSIM (0.96 ± 0.02) and PSNR (36.93 ± 3.93), underscoring the potential of GAN‐based approaches in medical imaging.

A key distinction lies in the clinical focus. While Bae et al. and Rani et al. emphasized chest radiography for nodule detection, our research targeted enhanced tumor localization for radiotherapy by removing bone shadows that obscure tumor boundaries. The superior SSIM and PSNR values observed confirm the efficacy of our GAN models in improving tumor visualization for treatment planning.

Existing methods struggle with tumor detection due to reduced visibility when tumors overlap with bony structures, degrading image quality. This study demonstrates that effective bone suppression preserves tumor contours and improves depiction accuracy by minimizing bone shadows. However, GAN‐based methods face challenges in reducing bone intensity and suffer from inherent training instability, including mode collapse, parameter divergence, and gradient vanishing. CUT addresses mode collapse and missing pixel issues through unsupervised learning, but DCLGAN improves upon this by maximizing mutual information and integrating CycleGAN with CUT. SimDCL further enhances performance by adding similarity loss, producing distinct and accurate outputs across various inputs. Han et al. showed that both CUT and DCLGAN produced nearly identical outputs regardless of input, whereas SimDCL generated distinct and accurate outputs for varying inputs [26]. Additionally, SimDCL consistently delivered high‐quality images, demonstrating its potential for automated brain MRI segmentation [45]. Table 2 and Figure 4 show the model's superiority, with SimDCL generating images that closely approximate reference images while mitigating mode collapse.

The ability of models like SimDCL to suppress bone shadows while preserving tumor structure integrity carries significant clinical relevance, particularly in radiotherapy planning. Precise tumor localization is crucial to ensure accurate radiation delivery and minimize damage to surrounding healthy tissues. The elevated SSIM and PSNR values observed in this study confirm that these models effectively preserve critical anatomical features, making them suitable for clinical use.

Furthermore, the low FID score of SimDCL (114.52 ± 32.60), indicating a high degree of realism in generated images, enhances its clinical utility. Realistic images are essential for radiologists and oncologists to accurately delineate tumor boundaries. The performance of CUT in template matching, demonstrated by a ZNCC value of 0.789 ± 0.159, further underscores its potential for real‐time tumor localization, particularly in image‐guided radiotherapy, where precision is paramount.

**FIGURE 5:**
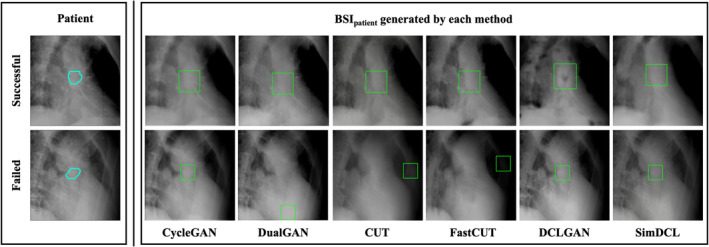
Template matching for each of actual treatment images and BSI_patient_. (L): The case with successful (upper) and failed (lower) detection of actual treatment images. (R): BSI_patient_ generated by each method. All images were adjusted to the window level 30 HU/window width 330 HU.

Figure 5 highlights certain instances where detection proved challenging, a phenomenon likely arising from the diverse clinical scenarios encountered during the study. Tumor variability in size, location, and respiratory motion emerged as pivotal factors affecting the accuracy and efficacy of bone‐suppressed image generation. These variations complicate the alignment between the region of interest and the template image, thereby impeding consistent and precise detection.

As shown in Table 3, CUT achieved the highest tumor identification accuracy. However, the lack of correlation between ZNCC values in Table 3 and the image quality metrics in Table 2 can be explained by several factors. Template matching relies heavily on template images, yet discrepancies in size, brightness, and contrast within the regions of interest can lead to significant deviations in detection outcomes, complicating the evaluation through metrics such as ZNCC. Oh et al. demonstrated that integrating Haar 2D wavelet decomposition with adversarial training yields superior outcomes, as a convolutional autoencoder incorporating wavelet decomposition generates images with diminished sharpness and contrast, thereby augmenting the model's overall performance [46]. While high ZNCC values indicate strong alignment with templates, they do not consistently align with superior perceptual image quality, as reflected in Table 2. This divergence arises because ZNCC is highly sensitive to minor content variations, whereas the comprehensive perceptual metrics in Table 2 evaluate broader image quality attributes. Consequently, despite SimDCL achieving optimal image quality, its tumor identification accuracy was inferior to that of CUT, which excelled in detection precision.

Additionally, common techniques in template matching involve utilizing residuals or normalized correlation as evaluation criteria. However, these methods face challenges related to misidentification stemming from changes in the image environment, and no definitive approach has been proposed. Common methods to prevent misidentification include normalized correlation [47], which eliminates image amplitude and offset, and incremental phase correlation [48], which matches based on the congruence of signs.

Despite the promising outcomes, this study has notable limitations. First, the models were assessed using phantom datasets, which may not entirely reflect the complexity inherent in clinical cases. These synthetic datasets lack the variability and noise characteristic of real‐world clinical data, thereby potentially inflating the perceived efficacy of the models. Future investigations should utilize larger, more heterogeneous clinical datasets to more accurately gauge the models' generalizability.

A further limitation pertains to the absence of explicit tumor selection criteria, particularly in challenging scenarios such as GGNs. In the absence of well‐defined criteria, comparing the performance of the models across various tumor types becomes problematic. Future studies should ensure the inclusion of a broad spectrum of tumor types, including GGNs, to facilitate a more thorough and representative evaluation.

Moreover, while SSIM and PSNR are valuable for assessing image quality, they fail to directly measure the accuracy of tumor detection. The inconsistent correlation with ZNCC indicates that the models may face challenges in tumor detection, particularly under varying conditions such as tumor size, location, and respiratory motion. It is essential that future research incorporates more appropriate metrics, such as the dice similarity coefficient, to provide a more precise assessment of detection performance.

Additionally, the study did not sufficiently account for the influence of respiratory motion, a factor that can significantly impact both image quality and detection accuracy in clinical practice. Future investigations should explore how respiratory motion affects model performance, as this is critical for the applicability of these models in real‐time clinical environments.

Finally, the study occasionally demonstrated inadequate noise suppression, leading to failures in tumor identification. Enhanced noise reduction techniques, coupled with clinically relevant metrics such as the dice similarity coefficient, are essential for improving the reliability of tumor detection in noisy images.

## 5 Conclusion

This study presents a GAN‐based method for bone suppression in fluoroscopic tumor images, enhancing tumor localization for radiotherapy while preserving image quality. These findings contribute to the growing body of research on GAN‐based bone suppression in medical imaging. However, challenges persist, notably the need to improve model robustness in clinical settings with variable respiratory motion. Expanding the evaluation to include clinical datasets is essential for validating these findings.

Future research should focus on integrating our models into real‐time radiotherapy workflows, aiming to further improve the precision and efficacy of cancer treatment and ultimately enhance patient outcomes. Our findings advance the field of medical imaging and pave the way for innovative applications of machine learning in clinical practice.

## Author Contributions

Conceptualization and design: Zennosuke Mochizuki and Masahide Saito. Radiological data acquisition: Zennosuke Mochizuki and Toshihiro Suzuki. Statistical analyses: Zennosuke Mochizuki and Masahide Saito. Manuscript writing and interpretation: Zennosuke Mochizuki and Masahide Saito. Final approval of manuscript: All authors.

## Ethics Statement

Ethical approval to report this case was obtained from * Ethics Committee of Kasugai General Rehabilitation Hospital [2023–1] *.

## Consent

Informed consent was obtained in the form of opt‐out on the website.

## Conflicts of Interest

The authors declare no conflicts of interest.

## Data Availability

The data that support the findings of this study are available from the corresponding author upon reasonable request.
